# Chloroplastic ATP Synthase Alleviates Photoinhibition of Photosystem I in Tobacco Illuminated at Chilling Temperature

**DOI:** 10.3389/fpls.2018.01648

**Published:** 2018-11-14

**Authors:** Ying-Jie Yang, Shi-Bao Zhang, Wei Huang

**Affiliations:** ^1^Key Laboratory of Economic Plants and Biotechnology, Kunming Institute of Botany, Chinese Academy of Sciences, Kunming, China; ^2^University of Chinese Academy of Sciences, Beijing, China

**Keywords:** chilling temperature, chloroplastic ATP synthase, proton motive force, ΔpH, photosystem I, photoprotection

## Abstract

Chloroplastic ATP synthase plays a significant role in the regulation of proton motive force (*pmf*) and proton gradient (ΔpH) across the thylakoid membranes. However, the regulation of chloroplastic ATP synthase at chilling temperature and its role in photoprotection are little known. In our present study, we examined the chlorophyll fluorescence, P700 signal, and electrochromic shift signal at 25°C, and 6°C in tobacco (*Nicotiana tabacum* L. cv. Samsun). Although photosynthetic electron flow through both PSI and PSII were severely inhibited at 6°C, non-photochemical quenching and P700 oxidation ratio were largely increased. During the photosynthetic induction under high light, the formation of *pmf* at 6°C was similar to that at 25°C. However, the ΔpH was significantly higher at 6°C, owing to the decreased activity of chloroplastic ATP synthase (*g*_H_^+^). During illumination at 6°C and high light, a high ΔpH made PSI to be highly oxidized, preventing PSI from photoinhibition. These results indicate that the down-regulation of *g*_H_^+^ is critical to the buildup of ΔpH at low temperature, adjusting the redox state of PSI, and thus preventing photodamage to PSI. Our findings highlight the importance of chloroplastic ATP synthase in photoprotection at chilling temperature.

## Introduction

Light drives photosynthesis in higher plants. However, excess excitation energy can induce photoinhibition in chloroplasts (Melis, 1999). Under environmental stresses, such as high-light intensities and drought conditions, photosystem II (PSII) is generally the most sensitive component whereas photosystem I (PSI) is relatively stable (Aro et al., 1993). However, at chilling-light stress, PSI is severely damaged, and the damage to PSII is often negligible in the chilling-sensitive plant cucumber (Havaux and Davaud, 1994; Sonoike and Terashima, 1994; Terashima et al., 1994; Barth and Krause, 1999). Photoinhibition of PSI severely affects linear electron flow (LEF), photosynthetic CO_2_ assimilation, photoprotection, and hence plant growth (Munekage et al., 2002, 2008; Suorsa et al., 2012, 2016; Brestic et al., 2015; Zivcak et al., 2015; Yamori et al., 2016). Furthermore, PSI recovers very slowly, which needs several days (Zhang and Scheller, 2004; Zivcak et al., 2015). Therefore, PSI photoinhibition has been regarded as an important reason for why some chilling-sensitive plants such as cucumber cannot survive at low temperature. At chilling-light stress, photoinhibition of PSI can be alleviated by the addition of methyl viologen that stimulates the oxidation of PSI reaction centers by accepting electrons from PSI (Sonoike et al., 1997; Barth and Krause, 1999). As a result, under such condition, PSI photoinhibition occurs when the PSI electron carriers are highly reduced. Tobacco is considered as a less chilling-sensitive species compared to cucumber (Barth and Krause, 1999). Specifically, PSI activity is less sensitive to low temperature associated with strong light in tobacco than in cucumber (Barth and Krause, 1999). However, mechanisms underlying the photoprotection of PSI at chilling-light stress in tobacco are not clear.

Recently, Miyake group reported that when electron carriers in PSI are highly reduced, excess light energy induced the production of reactive oxygen species (ROS) within the thylakoid membranes, and those ROS causes serious damage to PSI (Sejima et al., 2014; Takagi et al., 2016, 2017). Furthermore, the ROS-scavenging systems consisting of ascorbate peroxidase, and superoxide dismutase are insufficient to scavenge those ROS (Takagi et al., 2016). In order to prevent the production of ROS within the thylakoid membranes, PSI should be highly oxidized, diminishing the probability of electron donation from P700 to O_2_. The PSI redox state is mainly regulated by a proton gradient (ΔpH) across the thylakoid membranes (Yamamoto et al., 2016; Takagi et al., 2017). A higher ΔpH slows down the oxidation of PQH_2_ at Cyt *b_6_*/*f*, limiting the electron transfer to PSI, and thus contributing to the oxidation of P700 (Suorsa et al., 2012; Tikkanen and Aro, 2014; Tikkanen et al., 2015). As a result, the formation of a sufficient ΔpH is important to optimize the redox state of PSI, and prevent photoinhibition of PSI under environmental stresses.

In chloroplasts, the formation of ΔpH during photosynthesis is mainly dependent on two factors: (1) the accumulation of protons in the lumen from the water-splitting activity of PSII and from the electron transfer via Cyt *b_6_*/*f*, which relies on photosynthetic electron transport; (2) the efflux of H^+^ from lumen to the stromal side of thylakoid membranes (i.e., the activity of chloroplastic ATP synthase). In LEF, protons are released by water splitting in PSII and the quinone cycle in the Cyt *b*_6_/*f* complex, forming proton motive force (*pmf*) across the thylakoid membranes. During cyclic electron flow (CEF), electrons from either NADPH or ferredoxin are cycled back from PSI to the plastoquinone pool, generating a ΔpH without reduction of NADP^+^ (Johnson, 2011). In addition, chloroplastic ATP synthase controls the H^+^ efflux activity and thus plays a significant role in the formation of ΔpH (Kanazawa and Kramer, 2002; Rott et al., 2011; Kanazawa et al., 2017; Takagi et al., 2017). For example, at low CO_2_ concentration, the activity of chloroplastic ATP synthase is depressed to enhance ΔpH, modulating the thermal dissipation of excess light energy (Kanazawa and Kramer, 2002; Takagi et al., 2017). Under high light and fluctuating light, owing to the increased activity of chloroplastic ATP synthase, *Arabidopsis* mutants *hope2*, and *cfq* showed lower ΔpH than wild-type, resulting in severe photoinhibition of PSI and PSII (Takagi et al., 2017). As a result, coordination of photosynthetic electron flow, and chloroplastic ATP synthase regulate the ΔpH formation, and photoprotection. However, it is unclear whether alternative electron flow or chloroplastic ATP synthase is the critical component for the buildup of ΔpH at chilling-light stress.

At chilling temperature, alternative electron flows including CEF and water-water cycle are considered to have important roles in stimulating ΔpH formation (Hirotsu et al., 2004; Zhou et al., 2004; Huang et al., 2011, 2016b, 2017c). However, when illuminated at chilling temperature, the large decrease in electron flow through both PSI and PSII was accompanied with an increase in ΔpH for leaves of *Calotropis gigantea* (Huang et al., 2017c). As a result, the enhancement of ΔpH at chilling temperature cannot be explained by the changes in ETRI and ETRII. We hypothesize that the chloroplastic ATP synthase is probably the key determinant of ΔpH formation at chilling-light stress. In order to test this hypothesis, we examined the chlorophyll fluorescence, P700 signal, and electrochromic shift (ECS) signal at 25 and 6°C for leaves of a tobacco cultivar Samsun. Furthermore, the residual PSI and PSII activities after exposure to 1178 μmol photons m^-2^ s^-1^ at 6°C for 100 min were determined.

## Materials and Methods

### Plant Materials

In this study, we used tobacco (*Nicotiana tabacum* L. cv. Sumsan) to conduct experiments. This tobacco cultivar was chosen in particular because the PSI activity was insusceptible to low temperature and moderate light in it (Barth and Krause, 1999). Plants were cultivated in plastic pots in a phytotron with daily/night temperatures of 15/30°C and light condition of 95% sunlight. During the experimental period, plants were cultivated with sufficient water or nutrient. In the present study, mature, but not senescent leaves from 8-week-old plants were utilized for the experiments.

### Chlorophyll Fluorescence and P700 Measurements

Light response curves were monitored by simultaneously recording chlorophyll fluorescence and P700 redox state using the Dual PAM-100 (Heinz Walz, Effeltrich, Germany). In the present study, red light (635 nm) was used as actinic light. To generate light response curves, dark-adapted mature leaves were illuminated at 25°C and 1178 μmol photons m^-2^ s^-1^ for 15 min to activate photosynthetic sinks, followed by exposure to each light intensity (1178, 923, 611, 330, 172, 94 μmol photons m^-2^ s^-1^) for 2 min. Afterward, plants were transferred to 6°C, and the same leaves were illuminated at 611 μmol photons m^-2^ s^-1^ for 10 min, followed by measurements of light response curves as conducted at 25°C.

The chlorophyll fluorescence parameters were calculated as follows: *F*_v_/*F*_m_ = (*F*_m_ – *F*_o_)/*F*_m_, Y(II) = (*F*_m_′ – *F*_s_)/*F*_m_′ (Genty et al., 1989), non-photochemical quenching in PSII (NPQ) = (*F*_m_ – *F*_m_′)/*F*_m_′. *F*_o_ is the minimum fluorescence in the dark-adapted state. *F*_m_ and *F*_m_′ are the maximum fluorescence after dark-adapted and light-adapted, respectively. *F*_s_ is the light-adapted steady-state fluorescence. *F*_o_ and *F*_m_ were determined after dark adaptation for 30 min. The PSI photosynthetic parameters were measured according to the method of Klughammer and Schreiber (2008). The maximum photo-oxidizable P700 (*P*_m_) was determined to estimate the PSI activity (Huang et al., 2010a,b; Suorsa et al., 2012; Tikkanen et al., 2014; Yamori et al., 2016). The effective photochemical quantum yield of PSI was measured as Y(I) = (*P*_m_′ -*P*)/*P*_m_. The quantum yield of PSI non-photochemical energy dissipation due to donor side limitation was calculated as Y(ND) = *P*/*P*_m_. The quantum yield of non-photochemical energy dissipation due to the acceptor side limitation was measured as Y(NA) = (*P*_m_ - *P*_m_′)/*P*_m_.

The rate of photosynthetic electron transport was calculated as: ETRII = Y(II) × PPFD × 0.85 × 0.5, ETRI = Y(I) × PPFD × 0.85 × 0.5, where 0.5 is the proportion of absorbed light reaching PSI, or PSII, and 0.85 is the fraction of the incident light absorbed by leaves. The apparent rate of CEF was estimated as ETRI – ETRII (Huang et al., 2012, 2015, 2017b, 2018b; Zivcak et al., 2013), and the relative contribution of CEF to total electron flow was estimated as ETRI/ETRII ratio (Yamori et al., 2011, 2015).

### Electrochromic Shift (ECS) Analysis

The ECS signal was examined as the absorbance change at 515 nm by using a DUAL-PAM-100 (Walz, Effeltrich, and Germany) equipped with a P515/535 emitter-detector module (Walz). Plants were first dark adapted for 30 min to measure the 515 nm absorbance change induced by a single turnover flash (ECS_ST_). Afterward, we detected the ECS signal during photosynthetic induction at a high light of 1178 μmol photons m^-2^ s^-1^, and the slow relaxation of the ECS signal were analyzed after exposure for 15 min. Afterward, the ECS signal were recorded after exposure to each light intensity (1178, 923, 611, 330, 172, and 94 μmol photons m^-2^ s^-1^) for 2 min, during which 1-s dark pulse was applied to estimate the values of ECS_t_ and *g*_H_^+^ at each light intensity. The ECS dark interval relaxation kinetics (DIRK_ECS_) was analyzed according to the method of Sacksteder et al. (2001) and Takizawa et al. (2008), calculating *pmf* and *g*_H_^+^. The slow relaxation of the ECS signal is used to analyze ΔpH and the membrane potential (ΔΨ) across the thylakoid membranes. All ECS_t_ and ΔpH levels were normalized against ECS_ST_. This normalization accounted for changes in leaf thickness and chloroplast density between leaves (Takizawa et al., 2008; Wang et al., 2015). The activity of chloroplastic ATP synthase (*g*_H_^+^) was estimated as the inverse of the decay time constant [1/*τ*_ECS_] by fitting the first 300 ms of the decay curve with a first-order exponential decay kinetic (Sacksteder and Kramer, 2000; Cruz et al., 2005).

### Photoinhibitory Treatment

Before chilling-light treatment, whole plants were dark-adapted at 25°C for at least 30 min to measure *P*_m_ and *F*_v_/*F*_m_. Afterward, intact leaves were illuminated at 1178 μmol photons m^-2^ s^-1^ and 6°C for 100 min, and then values of *P*_m_ and *F*_v_/*F*_m_ were measured after dark adaptation for 30 min at 25°C.

### Statistical Analysis

We used independent *T*-test to detect differences between 25 and 6°C. All statistical analyses were conducted using SPSS 16.0 software.

## Results

### Effects of Chilling Temperature on *pmf* and the Activity of Chloroplastic ATP Synthase

**FIGURE 1:**
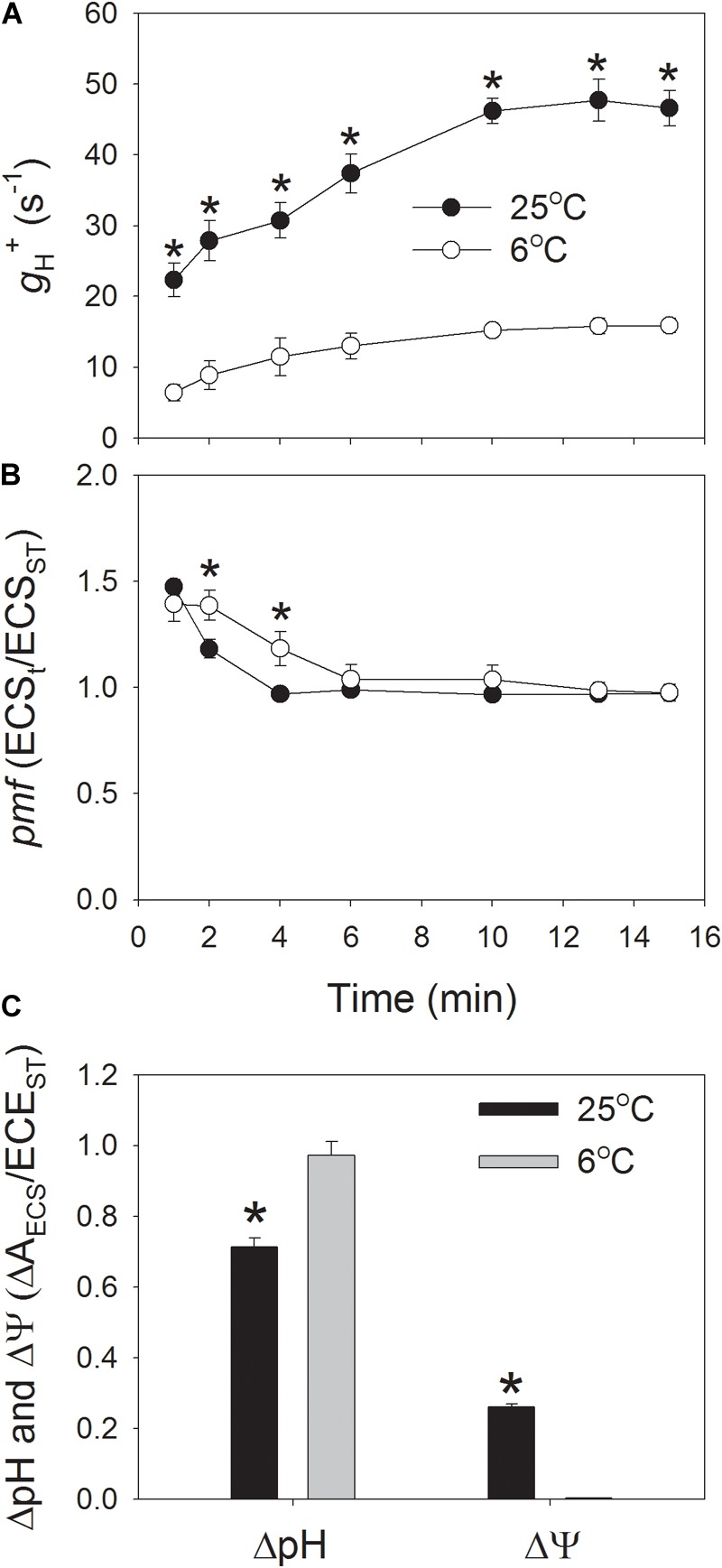
Fluctuations in the proton conductivity of the thylakoid ATP synthase (*g*_H_^+^) **(A)**, the proton motive force (*pmf*) **(B)**, and the proton gradient (ΔpH), and membrane potential (ΔΨ) **(C)** across the thylakoid membranes during the photosynthetic induction at 6 and 25°C. Plants were adapted to the dark at least 30 min before this analysis, and the actinic light was set at 1178 μmol photons m^-2^ s^-1^. After photosynthetic induction for 15 min, the ΔpH was analyzed. ECS_t_/ECS_ST_ represents the total *pmf* across thylakoid membranes, and *g*_H_^+^ indicates the activity of chloroplastic ATP synthase. Values are means ± SE (*n* = 4). Asterisks indicate a significant difference between 6 and 25°C.

In order to understand the regulation of *pmf* at low temperature, ECS signal was determined during photosynthetic induction at 1178 μmol photons m^-2^ s^-1^, and we analyzed *pmf* and proton conductance of chloroplastic ATP synthase (*g*_H_^+^). After onset of AL, *g*_H_^+^ was low and gradually increased to the maximum value at approximately 10 min (Figure 1A). During the whole phase of photosynthetic induction, the values of *g*_H_^+^ were significantly lower at 6°C when compared to 25°C (Figure 1A). After illumination at 1178 μmol photons m^-2^ s^-1^ for 15 min, the value of *g*_H_^+^ at 6°C was just 34% of that at 25°C, indicating that the activity of chloroplastic ATP synthase was largely depressed in tobacco leaves when chilled at high light. Because the activity of chloroplastic ATP synthase can significantly affect the buildup of *pmf*, the performance of *pmf* during photosynthetic induction was also monitored. When dark-adapted leaves were transferred to the high light, the *pmf* was rapidly formed in the first 1 min and gradually relaxed over time (Figure 1B). After exposure for 2 and 4 min, the values of *pmf* at 6°C were significantly higher than that at 25°C. However, during further induction phase, the total *pmf* did not differ significantly between 25 and 6°C (Figure 1B). The *pmf* is energetically composed of two components, ΔpH, and ΔΨ, and both them were analyzed after photosynthetic induction for 15 min. Interestingly, ΔpH was significantly increased but ΔΨ was largely depressed at the low temperature (Figure 1C). This result indicated that when tobacco leaves were chilled at high light, the thylakoid lumen became more acid although the total *pmf* did not change. These results were different from the *pmf* formation at low CO_2_ concentration. At a low CO_2_ concentration of 10 ppm, the decrease in ATP synthase activity led to the significant increase in *pmf* (Sukhov et al., 2016) Meanwhile, ΔpH was stimulated to favor photoprotection. However, ΔΨ was weakly influenced. Therefore, the partitioning of *pmf* into ΔpH at chilling temperature was probably regulated by the counter-ion fluxes across the thylakoid membrane.

**FIGURE 2:**
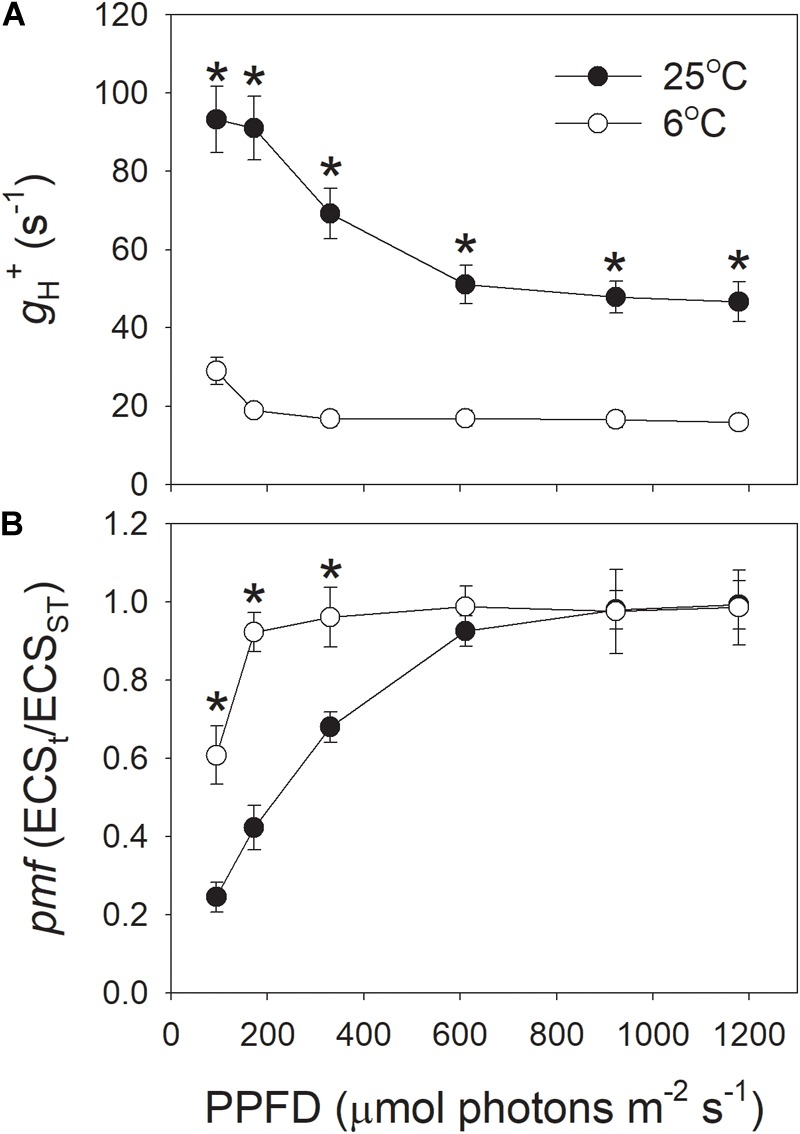
Light intensity dependence of the proton conductivity of the thylakoid ATP synthase (*g*_H_^+^) **(A)** and the *pmf* **(B)** across the thylakoid membranes at 6 and 25°C. Before this analysis, leaves were illuminated at 1178 μmol photons m^-2^ s^-1^ for 15 min to activate photosynthesis. Values for *g*_H_^+^ and *pmf* were obtained after exposure to each light intensity for 3 min. Values are means ± SE (*n* = 4). Asterisks indicate a significant difference between 6 and 25°C.

In addition, we examined the light intensity dependence of *g*_H_^+^ and *pmf* at 25°C, and 6°C (Figure 2). The results indicated that the values for *g*_H_^+^ under all light intensities were largely depressed by the low temperature of 6°C (Figure 2A), indicating the decreased activity of chloroplastic ATP synthase at 6°C, irrespective of the light intensity. Concomitantly, the total *pmf* was significantly enhanced under light intensities below 330 μmol photons m^-2^ s^-1^ (Figure 2B). These results suggested that the decrease in *g*_H_^+^ contributed to the enhancement of *pmf* at chilling temperature.

### Effect of Low Temperature on Photosynthetic Electron Flow

**FIGURE 3:**
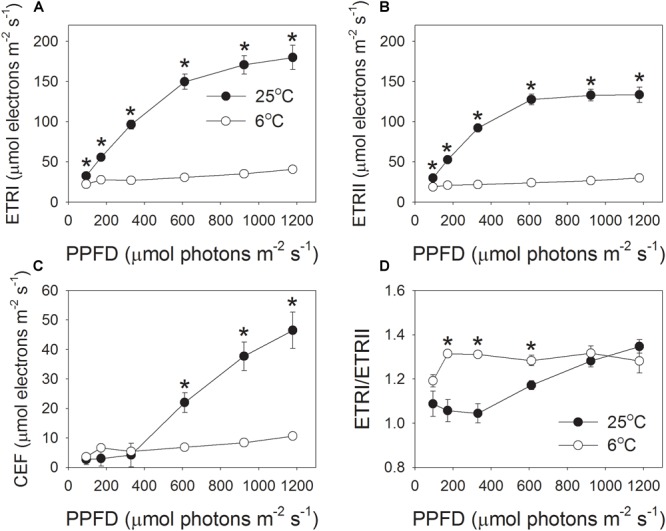
Light intensity dependence of the photosynthetic electron flow through PSI and PSII at 6°C and 25°C. **(A)** ETRI, electron transport rate through PSI; **(B)** ETRII, electron transport rate through PSII; **(C)** CEF, cyclic electron flow around PSI; **(D)** ETRI/ETRII ratio. Values are means ± SE (*n* = 4). Asterisks indicate a significant difference between 6°C and 25°C.

In order to understand the effect of low temperature on proton influx activity, the light intensity dependence of photosynthetic electron flow was measured at 6 and 25°C. The rates of electron flow through PSI and PSII were severely inhibited by the low temperature, especially under high light intensities (Figure 3). For example, at the high light of 1178 μmol photons m^-2^ s^-1^, values for ETRI at 25 and 6°C were 180 and 41 μmol electrons m^-2^ s^-1^, respectively, (Figure 3A). Concomitantly, values for ETRII were 133 (25°C) versus 30 μmol electrons m^-2^ s^-1^ (6°C) (Figure 3B). As a result, values for CEF at 1178 μmol photons m^-2^ s^-1^, estimated as ETRI minus ETRII, were 47 μmol–11 μmol electrons m^-2^ s^-1^, respectively, (Figure 3C). Interestingly, the values of ETRI, ETRII, and CEF at 1178 μmol photons m^-2^ s^-1^ decreased to approximately 23% when leaves were transferred from 25 to 6°C (Figure 3). Furthermore, the value of ETRI/ETRII ratio at this strong light did not differ significantly between 25 and 6°C (Figure 3D).

### Effects of Chilling Temperature on PSII Energy Quenching and PSI Redox State

Because the responses of PSI and PSII activities to excess light energy are significantly correlated to the redox state of electron transfer chains (Munekage et al., 2002, 2004; Suorsa et al., 2012, 2016; Brestic et al., 2016; Takagi et al., 2016; Yamori et al., 2016), the parameters related to PSII energy quenching and PSI redox state as a function of the incident light intensity were measured.

**FIGURE 4:**
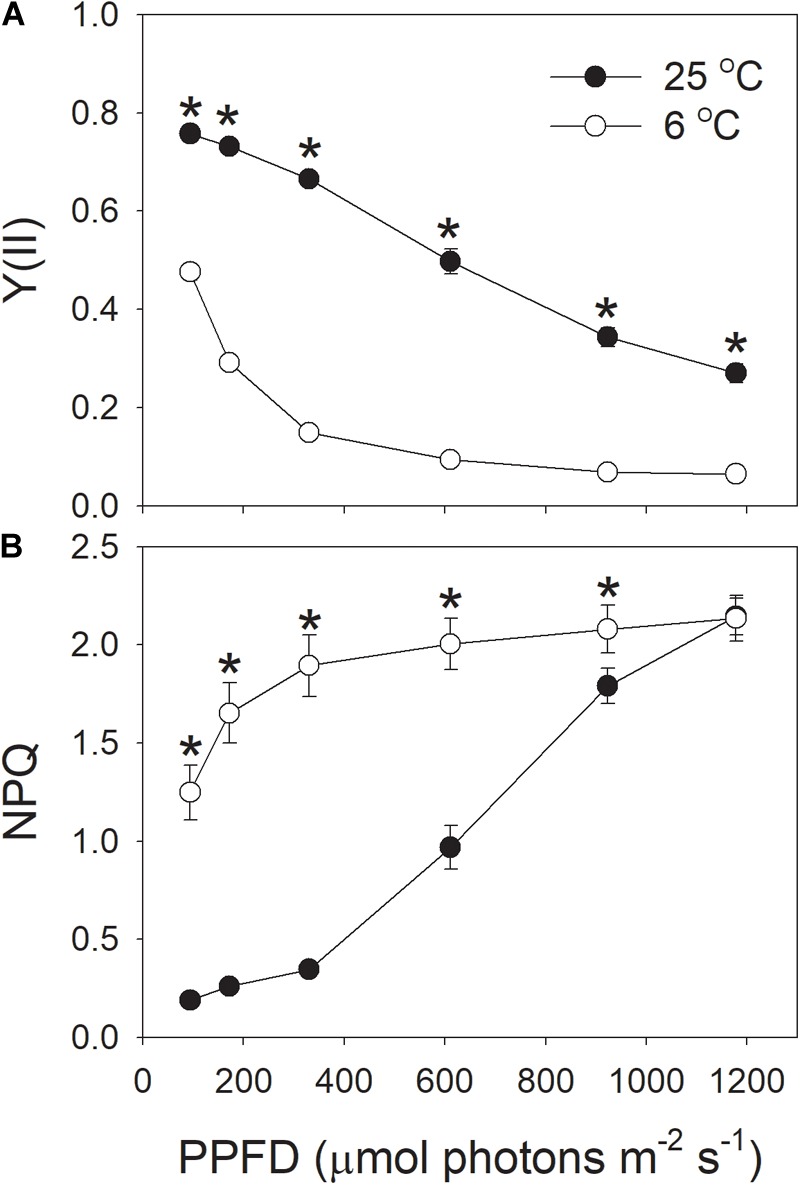
**(A,B)** Light intensity dependence of Y(II) (the effective quantum yield of PSII photochemistry) and NPQ (non-photochemical quenching in PSII) at 6 and 25°C. Values are means ± SE (*n* = 4). Asterisks indicate a significant difference between 6 and 25°C.

At the chilling temperature of 6°C, the quantum yield of PSII photochemistry [Y(II)] largely decreased under all light intensities, as compared to 25°C (Figure 4A), suggesting the decreased ability of plants to utilize the product of LEF, partly due to reduced Calvin-Benson cycle activity. This result was consistent with previous studies (Huang et al., 2011, 2016b, 2017c). Meanwhile, the NPQ was up-regulated to harmlessly dissipate excess light energy, especially at low light intensities (Figure 4B). At 6°C, NPQ was saturated at approximately 611 μmol photons m^-2^ s^-1^. At this light intensity, the value of NPQ at 25°C was half that at 6°C. At the high light of 1178 μmol photons m^-2^ s^-1^, the NPQ induction at 6°C was very similar to that at 25°C.

**FIGURE 5:**
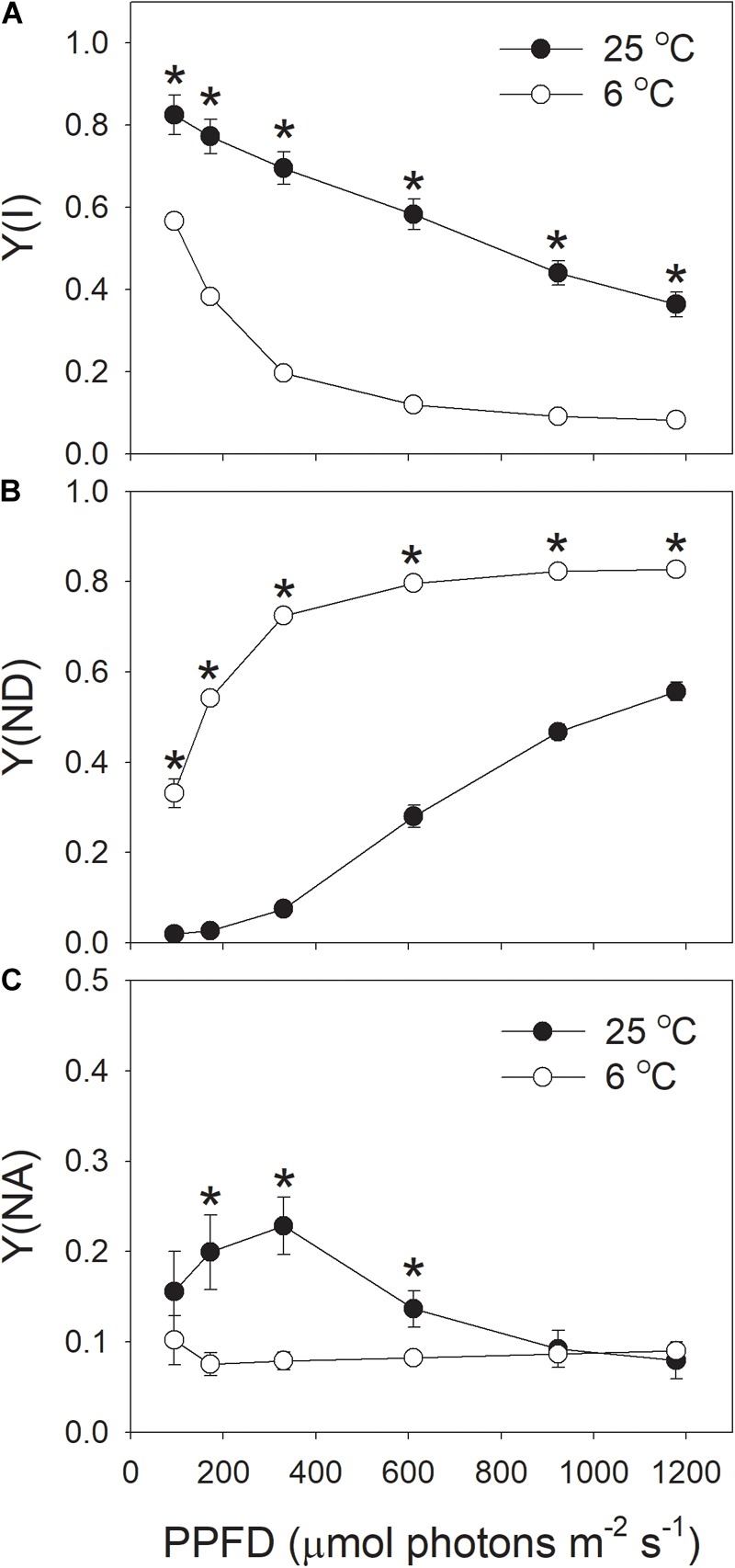
Light intensity dependence of PSI redox state at 6 and 25°C. **(A)** Y(I), the quantum yield of PSI photochemistry. **(B)** Y(ND), the quantum yield of PSI non-photochemical energy dissipation due to the donor-side limitation. **(C)** Y(NA), the quantum yield of PSI non-photochemical energy due to the acceptor-side limitation. Values are means ± SE (*n* = 4). Asterisks indicate a significant difference between 6 and 25°C.

The effective quantum yield of PSI [Y(I)] decreased gradually with increasing light intensity (Figure 5A), in accordance with previous reported results (Huang et al., 2011, 2017c; Kono et al., 2014). Similar to Y(II), Y(I) was largely inhibited by the low temperature (Figure 5A). With the increase in light intensity, the quantum yield of PSI non-photochemical quenching due to the donor side limitation [Y(ND)] gradually increased (Figure 5B), as expected from previous results reported in wild-type plants (Munekage et al., 2002, 2004; Kono et al., 2014). Furthermore, under all light intensities, values for Y(ND) were largely higher at 6°C than at 25°C, indicating that more P700 was in the oxidized state when leaves were illuminated at 6°C. The quantum yield of PSI non-photochemical quenching due to the acceptor side limitation [Y(NA)] was maintained at 0.1 when illuminated at 6°C (Figure 5C). By comparison, the value of Y(NA) at 25°C and high light was also maintained at 0.1 (Figure 5C). These results indicated that the over-reduction of electron carriers in PSI was prevented in these tobacco leaves illuminated at chilling temperature.

**FIGURE 6:**
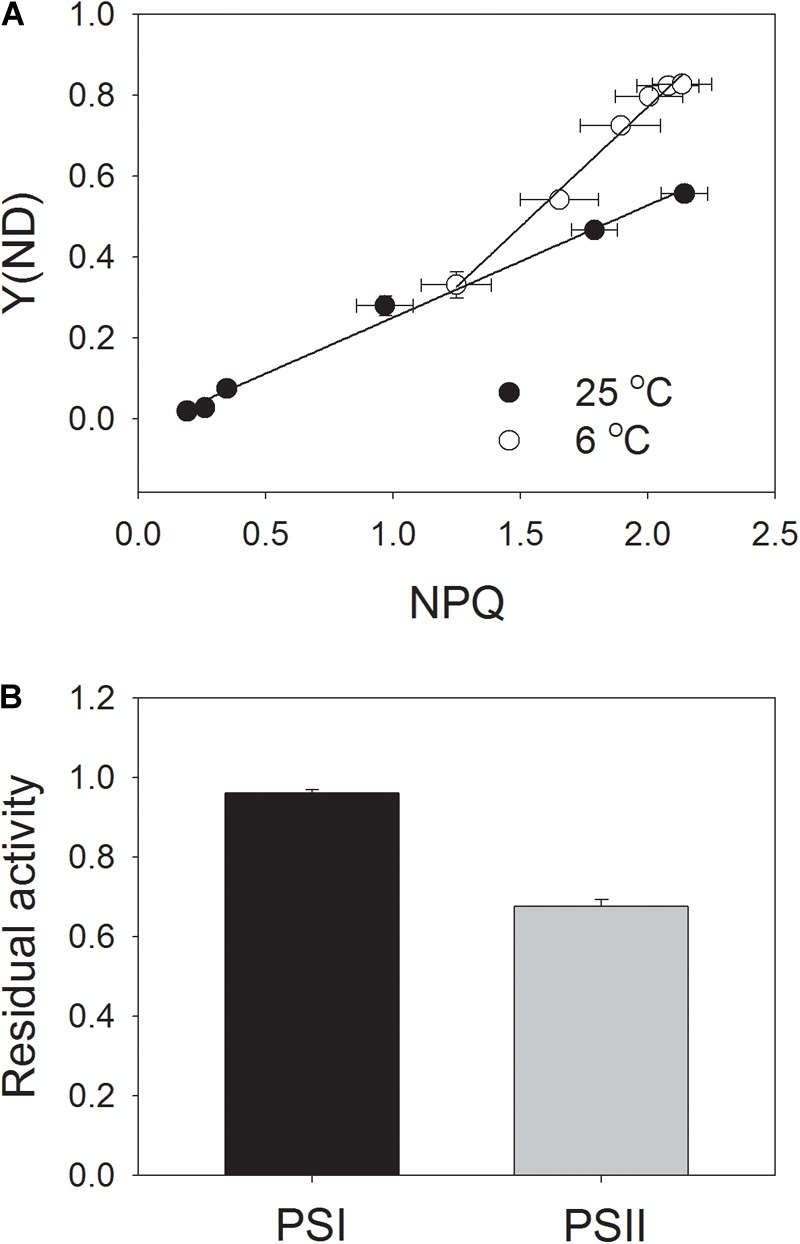
Correlation between NPQ and Y(ND) **(A)** and residual activity of PSI and PSII **(B)** after exposure to 1178 μmol photons m^-2^ s^-1^ at 6°C for 100 min. Data of Figures 4B, 5B were used to analyze the correlation between NPQ and Y(ND). After the chilling-light treatment, the *P*_m_ (maximum photo-oxidizable P700 content) and *F*_v_/*F*_m_ were measured, and the data were normalized to *P*_m_ and *F*_v_/*F*_m_ before treatment and are represented as the residual activity of PSII and PSI after chilling-light treatment. Values are means ± SE (*n* = 4).

Next, we analyzed the correlation between NPQ and Y(ND). We observed that, under high light, the same value of NPQ was accompanied with a higher Y(ND) at the chilling temperature (Figure 6A). This results suggested that the enhancement of ΔpH at chilling temperature was more important for oxidizing PSI than for inducing NPQ.

### Effect of Chilling Temperature on Photoinhibition

In order to understand the role of chloroplastic ATP synthase in photoprotection for PSI at chilling temperature, intact tobacco leaves were exposed to 6°C and 1178 μmol photons m^-2^ s^-1^ for 100 min, and the residual PSI and PSII activities were determined (Figure 6B). Interestingly, the value of *P*_m_ just decreased by 4% after this chilling-light treatment, indicating that PSI activity was not susceptible to this chilling-light stress. This result was consistent with the performance of PSI redox state as indicated in light response curves. By comparison, *F*_v_/*F*_m_ decreased by 32%, indicating a moderate photoinhibition of PSII.

## Discussion

### Tolerance of PSI Activity to Short-Term Chilling-Light Stress

Our results strongly indicated that, in leaves of the tobacco cultivar Samsun, the PSI activity hardly decreased after exposure to 6°C and 1178 μmol photons m^-2^ s^-1^ for 100 min (Figure 6B). Indeed, the tobacco cultivar Samsun is much less sensitive to chilling-light stress than the chilling-sensitive plant cucumber. For leaf disks of the tobacco cultivar Samsun, no inhibition of each photosystem was observed after 2 h illumination with 200 μmol photons m^-2^ s^-1^ at 4°C, but cucumber (*Cucumis sativus* L. cv. Mervita) leaf disks showed a decrease of 55% in PSI activity (Barth and Krause, 1999). These results suggest that the tobacco (cv. Samsun) leaves should have feasible mechanisms to prevent PSI against photoinhibition under chilling-light stress.

The mechanism of PSI photoinhibition under natural field conditions is dependent on plant species. A typical scheme of PSI photoinhibition proposes that the ROS produced within PSI cause PSI photoinhibition when electron carriers in PSI are highly reduced (Munekage et al., 2002; Takagi et al., 2016, 2017). However, ROS produced in the chloroplast stroma cause photoinhibition of PSI in some shade-establishing plants such as *Psychotria henryi, P. rubra*, and *Nephrolepis falciformis* (Huang et al., 2016c, 2017a, 2018c). At chilling temperature, light-induced photoinhibition of PSI could be alleviated by the addition of methyl viologen (Sonoike et al., 1997; Barth and Krause, 1999), which stimulates the production of ROS at the stromal region by accepting electrons from PSI. Therefore, a strong stromal sink prevents PSI photoinhbition. For the intact leaves of tobacco (*cv*. Samsun), the PSI reaction centers was highly oxidized at 6°C and 1178 μmol photons m^-2^ s^-1^ (Figure 5B). As a result, the probability of electron donation from P700 to O_2_ was suppressed, preventing the production of ROS in PSI and thus leading to the stability of PSI activity. In addition, PSII activity significantly decreased during the chilling-light treatment. Because PSII photoinhibition can decrease the electron transport to PSI and thus diminishes the production of ROS in PSI (Tikkanen et al., 2014; Huang et al., 2016a; Sukhov, 2016; Surova et al., 2016), the significant PSII photoinhibition may be another mechanism for protecting PSI against photoinhibition in tobacco leaves chilled at high light.

### *In vivo* Regulation of PSI Redox State at Chilling-Light Stress

Proton motive force plays a critical role in photoprotection for PSIthrough regulation of the redox state of PSI (Yamamoto et al., 2016;Shikanai and Yamamoto, 2017; Takagi et al., 2017). In order to understand why PSI was highly oxidized when the tobacco (*cv*. Samsun) leaves were chilled at high light, we determined the *pmf* during photosynthetic induction at 1178 μmol photons m^-2^ s^-1^ and 6°C. Interestingly, we found that high levels of *pmf* were observed during the photosynthetic induction at 6°C (Figure 1B). Furthermore, after induction for 15 min, the value of ΔpH was significantly higher at 6°C than at 25°C (Figure 1C). This high level of ΔpH controls electron transport to PSI, which involves two different mechanisms. Firstly, ΔpH slows down water splitting at the oxygen-evolving complex and induces NPQ to dissipate excessively absorbed light energy as heat from PSII antennae (Niyogi et al., 1998). The second is down-regulation of oxidation of PQH_2_ at Cyt *b_6_*/*f* complex, which contributes to oxidation of P700 when the acceptor side of PSI is open (Munekage et al., 2002, 2004; Suorsa et al., 2012, 2016; Yamamoto et al., 2016). These mechanisms control the rate of electron transfer from PSII to PSI, and prevent the over-reduction of electron carriers in PSI, protecting PSI activity under high light and fluctuating light (Munekage et al., 2002; Suorsa et al., 2012, 2016; Kono et al., 2014; Yamori et al., 2016). Once the formation of ΔpH was inhibited, PSI was very susceptible to high light and fluctuating light (Munekage et al., 2002; Suorsa et al., 2012; Tikkanen et al., 2014; Yamori et al., 2016; Kanazawa et al., 2017; Takagi et al., 2017). The light response curves indicated that the values of NPQ and Y(ND) were highly enhanced at the low temperature (Figures 4B, 5B), accompanying with high levels of *pmf* and low levels of *g*_H_^+^ (Figure 2). These results also suggested the upregulation of ΔpH at chilling-light stress. Therefore, the formation of an enhanced ΔpH under chilling-light stress optimized the redox state of P700 in PSI and minimized ROS production within PSI, thus preventing PSI photoinhibition.

### *In vivo* Regulation of Proton Motive Force at Chilling-Light Stress

Next, we examined the critical factor for the formation of an enhanced ΔpH at 1178 μmol photons m^-2^ s^-1^ and 6°C. The formation of ΔpH is determined by two factors: (i) the H^+^ influx activity in dependence on photosynthetic electron flow including LEF and CEF; and (ii) the H ^+^ efflux activity modulated by chloroplastic ATP synthase. We observed that both the LEF and CEF were largely depressed at chilling temperature (Figure 3). Furthermore, the ETRI/ETRII ratio at 1178 μmol photons m^-2^ s^-1^ did not change significantly between 25 and 6°C (Figure 3D), indicating that the chilling temperature hardly influenced the relative contribution of CEF to total electron transport at this high light. By comparison, ETRI/ETRII ratio was enhanced at light intensities below 611 μmol photons m^-2^ s^-1^ (Figure 3D). These results indicated that at low temperature CEF might play an important role in regulation of ΔpH at low and moderate light intensities but was less important at high light conditions. Usually, in some stress conditions such as drought, the high levels of NPQ under high light are accompanied with high levels of CEF (Huang et al., 2012; Zivcak et al., 2013, 2014), because the CEF-dependent generation of ΔpH can activate thermal energy dissipation. Now that the up-regulation of ΔpH at 1178 μmol photons m^-2^ s^-1^ and 6°C could not be explained by the changes in LEF and CEF, we paid attention to the rate of H ^+^ efflux from the lumen to stroma, which is managed by the chloroplastic ATP synthase. Chloroplastic ATP synthase significant affects ΔpH and thus regulates photosynthetic electron flow (Rott et al., 2011;Kanazawa et al., 2017; Takagi et al., 2017; Huang et al., 2018a). Once the activity of chloroplastic ATP synthase (*g*_H_^+^) is strongly repressed, the over-acidification of the thylakoid lumen restricts the assimilation capacity and LEF (Rott et al., 2011). Furthermore, once the *g*_H_^+^ is enhanced in *cfq* and *hope2* mutants of *Arabidopsis thaliana*, the formation of ΔpH is not sufficient, which subsequently causes photodamage to PSI and PSII (Kanazawa et al., 2017; Takagi et al., 2017). In *pgr5* mutant of *A. thaliana*, the increased *g*_H_^+^ impairs the buildup of ΔpH, leading to the over-reduction of PSI electron carriers and thus causing photoinhibition of PSI (Avenson et al., 2005; Suorsa et al., 2012, 2016; Wang et al., 2015; Shikanai and Yamamoto, 2017). As a result, *g*_H_^+^ is an important valve for photoprotection and plant growth. Interestingly, we here found that the *g*_H_^+^ values strongly decreased at the low temperature (Figures 1A, 2A), which restricted the rate of H^+^ efflux from thylakoid lumen to stroma. These results indicate that the chloroplastic ATP synthase, but not CEF, is critical to the buildup of an enhanced ΔpH at chilling temperature and high light, which provides new insight into the importance of chloroplastic ATP synthase in tolerance to low temperature.

At chilling temperature, the CO_2_ assimilation and photorespiration were extremely inhibited, based on the results of photosynthetic electron flow (Figure 3). The rate of CO_2_ assimilation can affect the modulation of *pmf* and ΔpH (Kanazawa and Kramer, 2002; Takagi et al., 2017). However, this process depends on the regulation of chloroplastic ATP synthase. In *Arabidopsis thaliana* mutants *pgr5* and *hope2*, the disturbed regulation of chloroplastic ATP synthase impaired the formation of *pmf* and ΔpH (Avenson et al., 2005; Takagi et al., 2017), causing photoinhibition of PSI under high light and fluctuating light. In chloroplasts, the activity of chloroplastic ATP synthase is thermodynamically regulated by the stromal ATP/ADP ratio. Takizawa et al. (2008) reported that *g*_H_^+^ was decreased by Pi deficiency in chloroplasts. Under conditions of high light and low temperature, the ATP/ADP ratio in chloroplasts increases due to the restriction of CO_2_ assimilation, leading to the decreased availability of ADP, and Pi. Consequently, the activity of chloroplastic ATP synthase was depressed, leading to lower values of *g*_H_^+^.

## Conclusion

In summary, our results indicate that chloroplastic ATP synthase plays a critical role in the regulation of ΔpH at chilling temperature and prevents PSI from photoinhibition. When plants are subjected to chilling-light stress, they are at risk of producing ROS in PSI. However, chloroplastic ATP synthase detects excess excitation energy by the slower ATP consumption rate or an unknown regulatory factor, and the decreased activity of chloroplastic ATP synthase contributes to the up-regulation of ΔpH. This high level of ΔpH slows down the electron transfer from PSII to PSI and avoids the over-reduction state in PSI, which would be beneficial for minimizing the production of ROS in PSI and preventing PSI photoinhibition. From the present study we propose that the chloroplastic ATP synthase, but not alternative electron flow, is critical for the formation of a sufficient ΔpH at low temperature and high light. Chloroplastic ATP synthase is a potential target to improve H^+^ efflux management and increase tolerance against low temperature stress under field conditions. Further study is needed to clarify the effect of impairment of *g*_H_^+^ regulation on PSI redox state and PSI photoinhibition at chilling-light stress.

## Author Contributions

WH and S-BZ designed the study. Y-JY and WH conducted the experiments. Y-JY, S-BZ, and WH analyzed the data. Y-JY wrote the manuscript with significant input from S-BZ and WH.

## Conflict of Interest Statement

The authors declare that the research was conducted in the absence of any commercial or financial relationships that could be construed as a potential conflict of interest.
